# Risk factors for mental disorders in women survivors of human trafficking: a historical cohort study

**DOI:** 10.1186/1471-244X-13-204

**Published:** 2013-08-03

**Authors:** Melanie Abas, Nicolae V Ostrovschi, Martin Prince, Viorel I Gorceag, Carolina Trigub, Siân Oram

**Affiliations:** 1 Institute of Psychiatry, Kings College London, London, UK; 2 N. Testemitanu Medical and Pharmaceutical University, Chisinau, Moldova; 3 United Nations Population Fund, Chisinau, Republic of Moldova; 4 ProGENEVA, Chisinau, Republic of Moldova

**Keywords:** Depression, PTSD, Human trafficking, Trauma, Violence, Women’s mental health

## Abstract

**Background:**

Previous studies have found high levels of symptoms of depression, anxiety, and post-traumatic stress disorder among women survivors of human trafficking. No previous research has described risk factors for diagnosed mental disorders in this population.

**Methods:**

A historical cohort study of women survivors of trafficked women aged 18 and over who returned to Moldova and registered for assistance with the International Organisation for Migration (IOM). Women were approached by IOM social workers and, if they gave informed consented to participate in the study, interviewed by the research team. At 2–12 months post-return to Moldova, a psychiatrist assessed DSM-IV mental disorders blind to information about women’s pre-trafficking and post-trafficking experiences using the Structured Clinical Interview for DSM-IV (SCID). A backwards stepwise selection procedure was used to create a multivariable regression model of risk factors for DSM-IV mental disorder measured at an average of 6 months post-return.

**Results:**

120/176 (68%) eligible women participated. At an average of 6 months post-return, 54% met criteria for any DSM-IV mental disorder: 35.8% of women had PTSD (alone or co-morbid), 12.5% had depression without PTSD and 5.8% had another anxiety disorder. Multivariable regression analysis found that childhood sexual abuse (Adjusted Odds Ratio [AOR] 4.68, 95% CI 1.04-20.92), increased number of post-trafficking unmet needs (AOR 1.80; 95% CI 1.28-2.52) and post-trafficking social support (AOR 0.64; 95% CI 0.52-0.79) were independent risk factors for mental disorder, and that duration of trafficking showed a borderline association with mental disorder (AOR 1.12, 95% CI 0.98-1.29).

**Conclusions:**

Assessment for mental disorders should be part of re-integration follow-up care for women survivors of human trafficking. Mental disorders at that time, most commonly PTSD and depression, are likely to be influenced by a range of predisposing, precipitating and maintaining factors. Care plans for survivors of trafficking must be based on individual needs, and must apply clinical guidelines for the treatment of PTSD and of depression. Evidence is needed on the effectiveness of therapy for PTSD in survivors of human trafficking.

## Background

Human trafficking is the recruitment and movement of people by force, coercion or deception, for the purposes of exploitation [1-4]. The global number of trafficked persons at any time is estimated to be 2.5 million [2]. Trafficked women and men are commonly exposed to severe trauma characterised by physical and sexual violence and threats while trafficked [5-9], and among those trafficked for sexual exploitation there is a high risk of HIV infection [8,10-12]. Evidence on the nature of psychological disorders among survivors of human trafficking is, however, very limited [8]. Previous studies have reported high levels of symptoms of depression, anxiety and post-traumatic stress disorder (PTSD) among women survivors of human trafficking, but have been hampered by using screening scales rather than a diagnostic instrument [7,13,14], by including women at different stages of trafficking, and by combining populations from different ethnicities, limiting internal validity [7]. No previous research has described risk factors for diagnosed mental disorder among women survivors of human trafficking.

The present study addresses this gap by assessing a consecutive sample of ethnic Moldovan women survivors of human trafficking assisted on return to Moldova using a diagnostic instrument to measure mental disorder [15] We previously reported that 54% of this sample met diagnostic criteria for mental disorder at an average of 6 months post-return to Moldova [16]. Given work in other trauma contexts we were particularly interested to consider abuse prior to trafficking and on post-trauma social stressors and social support as potential risk factors for diagnosed mental disorders in women survivors of human trafficking [15,17-19]. We hypothesized that:

a) childhood abuse would be associated with increased risk of mental disorder at an average of 6 months post-return, even after adjusting for pre-trafficking socio-economic position;

b) having more unmet social needs and less social support at an average of 6 months post-return would be associated with increased risk of mental disorder at that time, even after adjusting for baseline mental disorder measured immediately after return to Moldova.

## Methods

### Sampling and recruitment

Survey interviews were conducted between February 2008 and December 2008 with a consecutive sample of Moldovan women survivors of human trafficking. Women were eligible for inclusion if they were aged 18 or over, originally resident in Moldova, had returned to Moldova in the previous 12 months following a trafficking experience outside Moldova, had registered with the International Organisation for Migration (IOM) Assistance and Protection Programme (APP) in Moldova as a survivor of trafficking, and had accessed IOM crisis-intervention care for at least one day upon return. IOM defines human trafficking in accordance with the United Nations Protocol to Prevent, Suppress and Punish Trafficking in Persons [4]. All women survivors of human trafficking who are assisted to return to Moldova are eligible to receive IOM crisis-intervention care. Crisis-intervention care includes an assessment of medical, psychological, legal and social needs and the provision of residential care for up to 1 month [16,20]. Crisis-intervention care is followed by up to 12 months community rehabilitation for women who opt to receive further post-trafficking support from IOM [16,20] Approximately 80% of women survivors of human trafficking who are assisted to return to Moldova by the IOM access support from the APP programme; between 2000 and 2008 the IOM supported 2,340 women survivors of human trafficking who returned to Moldova. Women were excluded from this research if the IOM social worker or researcher considered them to be too distressed or unwell to participate. Trafficked men were excluded from this study because the programme is funded to provide post-trafficking support to women and children only.

### Ethics and consent

An IOM social worker approached women first and informed them of the study aims and subject matter, and emphasised the voluntary nature of participation and explained to women that their support would be in no way affected by their decision to participate, or not participate in the study. Women who consented to be approached by the research team were then contacted by the interviewers. When approaching a potential participant, the interviewers first introduced themselves and showed an identification card, and then explained the study aims, the subject matter, and the organizations involved. Women giving informed consent to participate were interviewed by the researcher at the IOM Rehabilitation Centre or another place of their choosing. In most cases women chose to be interviewed when they attended the IOM Rehabilitation Centre for their monthly review with their social worker. Travel expenses were reimbursed for women who chose to be interviewed at other times; there was no payment for participation in the study. For safety reasons, interviewers worked in teams of two if interviews were conducted in the community.

Survey interviews were conducted by two female interviewers who had experience of working with women survivors of human trafficking at IOM and had an undergraduate background in psychology. Interviewers received 6 days training in how to conduct the interview and assessments; sensitive and ethical issues; Moldova’s legal framework for responding to human trafficking; and women survivors’ rights of confidentiality, protection assistance and rehabilitation. Training included seminars, 2 days of simulated interviews, and 8 pilot interviews per interviewer. We followed the World Health Organization Ethical and Safety Recommendations for Interviewing Trafficked Women and complied with IOM data protection principles [21,22].

Following discussions with IOM it was considered that women may be distressed by recounting their trafficking experiences, including with regards to how they had been trafficked, threats to themselves and others, witnessing and experiencing physical and sexual violence, sustaining injuries, and leaving the trafficking situation. As the primary aim of the study was to describe the mental health status of women survivors of human trafficking, we therefore chose not to ask about the trafficking experience itself. Instead, we made use of existing data on the duration and type of trafficking and the country to which women had been trafficked, which had been collected by IOM during APP registration. Researchers were, however, trained to listen sensitively and non-judgmentally to women if they chose to disclose information about their experiences while trafficked, to emphasize that they were not to blame, and encourage them to speak with their support worker. Ethical approval was obtained from Kings College Research Ethics Committee (CREC/07/08-56) and from N. Testemitanu State Medical and Pharmaceutical University Institutional Review Board.

### Measures

#### Pre-trafficking

##### Demographic data

Data from routine IOM records completed during registration with the APP in Moldova provided information on pre-trafficking employment status, current marital status, and current age.

##### Childhood abuse

Childhood abuse was assessed on average 6 months post-return (range 2 to 12 months) to Moldova, using a self-administered questionnaire previously used with migrants [23]. The questionnaire comprised modified versions of the physical and emotional subscales of the Conflict Tactic Scale (CTS). Questions from the 10-item physical abuse subscale included “before the age of 17, did a parent or another adult in the household (i) spank you on the bottom with a bare hand; (ii) hit you on the bottom with something like a belt, hairbrush, stick or other hard object; (iii) slap you on the face, head or ears?” The 6 item emotional abuse subscale included questions such as “before the age of 17, did a parent or another adult in the household (i) shout, yell or scream at you; (ii) said that he/she would send you away.” The questionnaire also included two items on sexual abuse: “before the age of 17, did an adult or older child (i) touch you in a sexual way; (ii) force you to have any type of sexual intercourse?” [24,25]. If women were trafficked before the age of 17, they were asked if they had experienced abuse prior to being trafficked. Elsewhere, the CTS has demonstrated stable factor structure with satisfactory internal consistency, and has been shown to be valid cross-culturally [24,26]. Cronbach’s alphas for the 10 physical abuse, 6 emotional abuse, and 2 sexual abuse items were 0.88, 0.88, and 0.70, respectively. Items were translated and adapted following focus group discussions with local key informants.

### Trafficking

Data on country trafficked to, duration of trafficking in destination country, type of exploitation, and time since return to Moldova were collected by IOM during women’s registration with the APP programme in Moldova.

#### Post-trafficking

##### Mental disorder

Assessments of participants’ psychiatric condition upon return to Moldova were made available to the research team by IOM. These initial assessments were conducted within two days of women’s arrival at IOM by a senior consultant psychiatrist, based on the International Classification of Diseases (ICD-10) [27]. Full details are reported elsewhere [16]. For the purposes of this study, presence of baseline mental disorder was analysed as a binary variable.

During survey interviews, conducted on average 6 months post-return to Moldova (range 2–12 months), participants were assessed for current (last month) DSM-IV Axis I mental disorder by a Moldovan psychiatrist. Assessments were conducted using the official translated Romanian Non-Patient version of the Structured Clinical Interview for DSM–IV Axis I Disorders (SCID) [28,29]. This version was pre-tested by two Moldovan psychiatrists trained in standardized interviews. It was understandable to participants and had good face validity. When assessing PTSD, rather than asking women in detail about their experiences while trafficked, the psychiatrist asked “while women are in the trafficking situation, it is common to go through very difficult experiences, such as being seriously harmed or injured, or being threatened that you or someone close to you will be seriously harmed, or another type of very horrible experience: did anything like this happen to you?” If they reported yes, the interviewer proceeded to ask about PTSD symptoms in relation to this event; this approach meets accepted methods [30].

### Social stressors

Current social stressors were measured on average 6 months post-return to Moldova (range 2–12 months) using a modified version of the Camberwell Assessment of Need Short Appraisal Schedule (CANSAS-SF) [31], which has been widely used internationally to assess the needs of people with psychological disorders. Correlations of inter-rater reliability by service users and test-retest reliability have been shown elsewhere to be very high (r=0.98, P<0.01 and r=0.71, P<0.001 respectively) [31]. The original questionnaire contains 22 needs grouped into basic (e.g. accommodation, food), health (mental and physical), social, functioning and access to services. Following formative work, we added employment, safety from others, legal assistance, and self-esteem. Items on mental health or social support were dropped for the purposes of the presented analysis as they were rated elsewhere in the questionnaire; the final scale consisted of 17 items (α=0.82). Based on women’s reports of their current level of difficulty in relation to each item and the help they were receiving for that issue, items were scored by trained interviewers as either posing no serious problem; no or moderate problem due to help given; serious problem; or not known. Higher scores reflect greater levels of perceived current social stressors.

The strength of women’s social support networks was measured using the Duke Functional Social Support Questionnaire [32]. The questionnaire includes three items relating to affective support (such as “I get love and affection”) and five items relating to confidante support (such as “I get chances to talk to someone I trust about my personal and family problems”), each scored from 1 to 5 ranging from *much less than I would like* (scored as 1) to *as much as I would like* (scored as 5) [33]. Higher scores reflect greater levels of perceived social support. Following local discussions, we substituted the affective support item ‘*I get help when I am sick in bed’* for ’I *get help when I am in trouble*’. Cronbach’s alphas of 0.64 and 0.62 have been reported elsewhere for the three item affective support subscale and the five item confidante support subscale respectively [33]. In this study, Cronbach’s alphas of 0.04 and 0.09 were recorded for the affective support and confidante support subscales, respectively. As the Duke Functional Social Support Questionnaire subscales achieved unsatisfactory reliability scores in this sample, analyses used the total social support score (i.e. the sum of the eight items). Cronbach’s alpha for the sum scale was 0.12.

### Statistical analysis

Analyses used SCID-diagnosed DSM-IV mental disorder as the dependent variable of interest; the study was not adequately powered to conduct analyses by type of DSM-IV mental disorder. In the first stage of analysis, we used basic descriptive techniques and chi square tests to describe the frequency and distribution of exposure variables and their bivariate associations with DSM-IV mental disorder. Fisher exact tests were used when cell counts were lower than 5 [34]. Continuous variables were analysed using bivariate logistic regression. In the second stage of analysis, and to test our hypotheses, we used logistic regression to calculate adjusted odds ratios describing the relationship between DSM-IV mental disorder and (a) emotional, physical and sexual childhood abuse (three analyses, each adjusting for socio-economic position); and (b) unmet needs and social support score (two analyses, each adjusting for baseline mental disorder measured immediately after return to Moldova). Education level and pre-trafficking employment status were used as proxy variables when controlling for socio-economic position; due to small cell counts, these variables were recoded as binary variables during regression analyses. Fisher exact tests using these binary variables showed that their association with mental disorder was of comparable statistical significance to that of the original categorical variables. Finally, a multivariable regression model of risk factors for DSM-IV mental disorder at an average of 6 months post-return was created using a backwards stepwise selection procedure. Pre-trafficking, trafficking, and post-trafficking variables which showed an association with mental disorder in univariable analyses (p<0.1) were considered in the backwards stepwise selection procedure. Exposure variables were retained in the model if the covariate showed an association (p<0.1) with DSM-IV mental disorder measured at an average of 6 months post-return, when adjusted for the other retained covariates. Backwards stepwise selection was used because of the exploratory nature of the analysis and also because high levels of collinearity within the dataset precluded the creation of a model that included all relevant variables. All analyses were conducted using STATA 11 [35].

## Results

**Figure 1:**
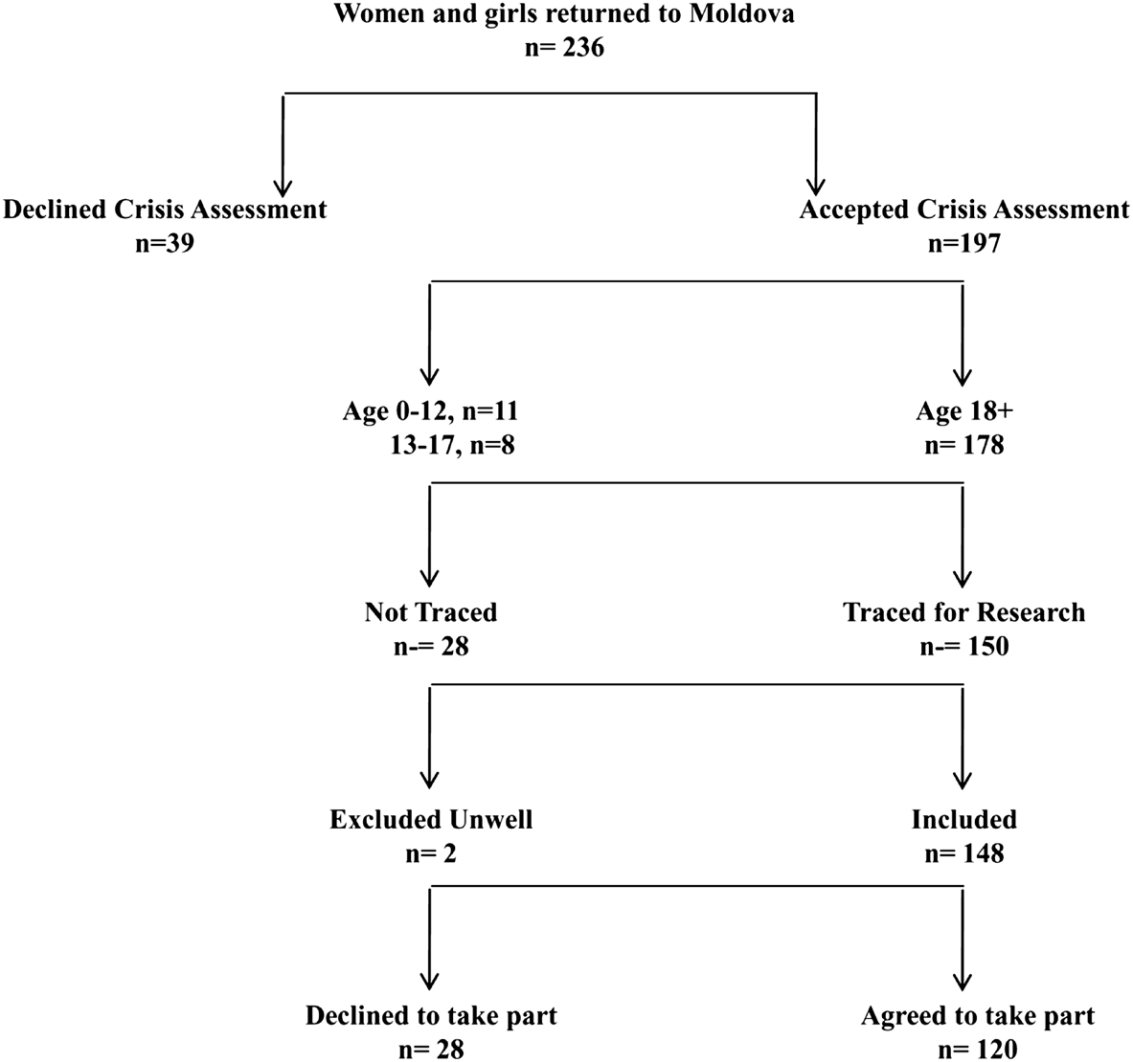
**Recruitment of women into the study from all the women and girls who returned to Moldova through IOM Assistance and Protection services from December 2007 to December 2008.** Originally published in Ostrovschi et al. [16] (reproduced with permission).

As shown in Figure 1, 216 women survivors of human trafficking returned to Moldova via the IOM APP program during the study period. 178 met the study inclusion criteria, of whom 2 were excluded due to severe ongoing physical illnesses. Of the 176 women, 28 could not be traced by IOM social workers, 9 declined to be approached by the research team following contact with the IOM social worker, and 19 declined to give informed consent upon contact with the research team. 120/176 (68%) women completed interviews at a mean of 6 months post-return (range 2–12 months). IOM provided restricted access to anonymized data to enable broad comparisons to be made between participants and non-participants. No significant differences were observed in respect of age, country trafficked to, duration of trafficking, marital status, or pre-trafficking employment status [16].

### Characteristics of women survivors of human trafficking

As shown in Table 1, the women in our sample ranged from age 18 to 44 years (mean 25.4, SD 6.0). Women had been trafficked to Turkey (39.7%), Russia (27.5%), the European Union (11.6%) and elsewhere (21.2%), including Bosnia and Herzegovina, Croatia, Israel, Kosovo, Serbia, Ukraine, and the United Arab Emirates. 11.7% were married or cohabiting at the time of interview and 51.7% had living children. Only a quarter (25.8%) had completed upper secondary education (from age 16 to 18) or higher. The majority of women (80.8%) had been trafficked for sexual exploitation. The average duration of trafficking was 9.6 months (SD 5.6, range 2–31 months). During the earlier baseline assessments, conducted when women had first returned to Moldova, 85.0% met criteria for mood or anxiety disorder.

**Table 1:** Comparison of characteristics of women survivors of human trafficking with and without mental disorder at an average of 6 months post-return: univariable analyses (n=120)*

		**Total (n=120)**	**No mental disorder (n=55)**	**Mental disorder(n=65)**	**p value**
		**N (%)**	**N (%)**	**N (%)**	
**Pre trafficking factors**					
Education status	General obligatory or less (age 6–15 years)	89 (74.2)	32 (58.2)	57 (87.7)	<.0.001
	Upper secondary or higher (age 16+ years)	31 (25.8)	23 (41.8)	8 (12.3)	
Employment status	Unemployed	82 (68.3)	37 (67.3)	45 (69.2)	0.009
	Unqualified work	25 (20.8)	7 (12.7)	18 (27.7)	
	Student	7 (5.8)	6 (10.9)	1 (1.5)	
	Qualified work	6 (5.0)	5 (9.1)	1 (1.5)	
Living situation	Alone	50 (41.2)	20 (36.4)	30 (46.2)	0.187
	With parents	21 (17.5)	7 (12.7)	14 (21.5)	
	With child(ren) only	25 (20.8)	14 (25.5)	11 (16.9)	
	With partner	13 (10.8)	6 (10.9)	7 (10.8)	
	Other	11 (9.2)	8 (14.6)	3 (4.6)	
Residence	Rural	81 (67.5)	32 (58.2)	49 (75.4)	0.045
	Urban	39 (32.5)	23 (41.8)	16 (24.6)	
Had a confidante	No	69 (57.5)	34 (61.8)	35 (53.9)	0.379
	Yes	51 (42.5)	21 (38.2)	30 (40.1)	
Childhood emotional abuse**	No	34 (28.3)	23 (41.8)	11 (16.9)	0.003
	Yes	86 (71.7)	32 (58.2)	54 (83.1)	
Childhood physical abuse* *	No	41 (34.2)	28 (50.9)	13 (20.0)	<0.001
	Yes	79 (65.8)	27 (49.1)	52 (80.0)	
Childhood sexual abuse	No	83 (69.2)	45 (81.8)	38 (58.5)	0.006
	Yes	37 (30.8)	10 (18.2)	27 (41.5)	
**Trafficking characteristics**					
Destination country	Turkey	46 (38.3)	25 (45.5)	21 (32.3)	0.286
	Russia	34 (28.3)	15 (27.3)	19 (29.2)	
	Other	40 (33.3)	15 (27.3)	25 (38.5)	
Type of exploitation	Labour	23 (19.2)	12 (21.8)	11 (16.9)	0.497
	Sexual	97 (80.8)	43 (78.2)	54 (83.1)	
Duration of trafficking (months)	Mean (SD, range)	9.6 (SD 5.6, range 2–31)	8.3 (SD 5.0, range 2–25)	10.7 (SD 6.0, range 2–31)	0.023
**Post trafficking baseline mental disorder (within 2 days of return to Moldova)**					
Baseline diagnosis of mental disorder	No	18 (15.0)	9 (16.4)	9 (13.9)	0.700
	Yes	102 (85.0)	46 (83.6)	56 (86.1)	
**Post trafficking factors (6 months after return to Moldova)**					
Age (years)	Mean (SD)	25.4 (6.0)	26.3 (6.5)	24.6 (5.4)	0.114
Marital status	Single/widowed/ divorced	106 (88.3)	42 (76.4)	64 (98.5)	<0.001
	Married/ cohabiting	14 (11.7)	13 (23.6)	1 (1.5)	
Has children	No	58 (48.3)	25 (45.5)	33 (50.8)	0.562
	Yes	62 (51.7)	30 (54.5)	32 (49.2)	
Employment status	Unemployed	44 (36.7)	10 (18.2)	34 (52.3)	<0.001
	Unqualified work	37 (30.8)	14 (25.5)	23 (35.4)	
	Student	25 (20.8)	18 (32.7)	7 (10.8)	
	Qualified work	14 (11.7)	13 (23.6)	1 (1.5)	
Time since return to Moldova (months)	Mean (SD, range)	5.9 (SD 3.2, range 2–12)	5.4 (SD 3.3, range 2–12)	6.3 (SD 3.3, range 2–12)	0.131
Number of unmet needs	Mean (SD, range)	3.7 (SD 2.8, range 0–11)	1.6 (SD 1.7, range 0–8)	5.4 (SD 2.3, range 1–11)	<0.001
Social support score	Mean (SD, range)	19.9 (SD 5.5, range 9–30)	24.3 (SD 3.5, range 16–30)	16.1 (SD 3.7, range 9–30)	<0.001

* Categorical variables were tested using chi-squared tests. Fisher exact tests were used when cell counts were lower than 5, Continuous variables were analysed using logistic regression.

** Abuse prior to age 17 by a parent or other adult in the household.

Women reported an average of 3.8 unmet needs (SD 2.8, range 0–11); the most commonly reported were difficulties with daily activities (51.7%), accommodation (42.5%), employment (40.0%), and lack of money (39.2%). 68.3% of women reported that they had been unemployed prior to trafficking. 36.7% of were currently unemployed. Over three quarters (79.2%) reported abuse in childhood; 30.8% reported sexual abuse, 65.8% physical abuse, and 71.7% emotional abuse.

54.2% of women in the sample were diagnosed with mental disorder (Table 2). The most common diagnoses were PTSD, depressive or other anxiety disorder; 35.8% of women had PTSD (alone or co-morbid), 12.5% had depression without PTSD and 5.8% had another anxiety disorder.

**Table 2:** Mental disorder among women survivors of human trafficking at an average of 6 months post-return (n=120)

	**Mental disorder (DSM-IV) n (%)**
Any DSM-IV mood or anxiety disorder	65 (54.2)
Posttraumatic stress disorder alone (PTSD)	18 (15.0)
PTSD co-morbid with depressive, other anxiety, or substance use disorder	25 (20.8)
Depressive disorder alone or co-morbid with substance use disorder	15 (12.5)
Other anxiety disorder (not PTSD) alone or co-morbid with substance use disorder)	7 (5.8)

### Risk factors for mental disorder

Univariable predictors of mental disorder at an average of 6 months post-return to Moldova included education status; pre-trafficking employment status; pre-trafficking residence (rural or urban) childhood emotional abuse, physical abuse, and sexual abuse; duration of trafficking; post-trafficking marital status; post-trafficking employment status; number of unmet needs; and social support score (Table 1). Type of exploitation (sexual versus labour), mental disorder at baseline, and time since returning to Moldova (months) did not show an association with mental disorder in univariable analyses.

As shown in Table 3, the risk of mental disorder at an average of 6 months post-return was increased by childhood emotional abuse (AOR 3.30; 95% CI 1.36-7.99), physical abuse (AOR 3.82; 95% CI 1.63-8.97) and sexual abuse (AOR 3.66; 95% CI 1.45-9.20) even after adjusting for pre-trafficking socio-economic position.

**Table 3:** Association between mental disorder at an average of 6 months post return and (1) childhood emotional abuse, (2) childhood physical abuse, and (3) childhood sexual abuse among women survivors of human trafficking (n=120)

**Child abuse**		**Odds ratio (95% CI)**	**p value**	Adjusted odds ratio* (95% CI)	**p value**
Analysis 1) Emotional abuse**	No	1		1	
	Yes	3.53 (1.52–8.18)	0.003	3.30 (1.36–7.99)	0.008
Analysis 2)	No	1		1	
Physical abuse**	Yes	4.15 (1.85–9.28)	0.001	3.82 (1.63–8.97)	0.002
Analysis 3) Sexual abuse	No	1		1	
	Yes	3.20 (1.37–7.44)	0.007	3.66 (1.45–9.20)	0.006

* All three analyses adjusted for education and pre-trafficking employment only (i.e. abuse variables are not adjusted for each other).

** Abuse prior to age 17 by a parent or other adult in the household.

The risk of mental disorder at an average of 6 months post-return was also increased by having more unmet needs (AOR 2.21; 95% CI 1.71-2.84) and decreased by having higher levels of social support (AOR 0.61; 95% CI 0.52-0.71), even after adjusting for baseline mental disorder assessed when women had first returned to Moldova (Table 4).

**Table 4:** Association between mental disorder at an average of 6 months post-return and (1) social support and (2) unmet needs among women survivors of human trafficking (n=120)

	**Odds ratio (95% CI)**	**p value**	Adjusted odds ratio* (95% CI)	**p value**
Analysis 1) Social support score	0.61 (0.52–0.72)	<0.001	0.61 (0.52–0.71)	<0.001
Analysis 2) Number of unmet needs	2.21 (1.71–2.85)	<0.001	2.21 (1.71–2.84)	<0.001

* Both analyses adjusted for baseline mood or anxiety disorder only (i.e. social support score and number of unmet needs are not adjusted for each other).

Eleven variables were associated (p<0.1) with mental disorder at an average of 6 months post-return in univariable analyses. A multivariable regression model was created using a backwards stepwise selection procedure which considered ten of these variables; post-trafficking marital status was not included in the backwards stepwise selection procedure because its effects were seen to be driven by very small numbers (see Table 1). The subsequent multivariable regression model retained four variables, which remained significant at p<0.1 whilst adjusted for the other retained covariates. Significant independent risk factors for mental disorder at an average of 6 months post-return included: childhood sexual abuse (AOR 4.68, 95% CI 1.04-20.92); social support score (AOR 0.64; 95% CI 0.52-0.79); and number of unmet needs (AOR 1.80; 95% CI 1.28-2.52) (see Table 5). Duration of trafficking showed a borderline association with mental disorder (AOR 1.12, 95% CI 0.98-1.29).

**Table 5:** Multivariable regression model of risk factors for mental disorder at an average of 6 months post-return among women survivors of human trafficking (n=120)*

**Pre-trafficking, trafficking and post-trafficking exposures**		**Adjusted odds ratio (95% CI)**	**p value**
Sexual abuse	No	1	
	Yes	4.67 (1.04–20.92)	0.044
Duration of trafficking (months)		1.12 (0.98–1.29)	0.089
Social support score (range 9–30)		0.64 (0.52–0.79)	<0.001
Number of unmet needs (range 0–11)		1.80 (1.28–2.52)	0.001

* Multivariable regression model using a backwards stepwise selection procedure considering the following variables: education status, pre-trafficking employment status, urban/rural residence, childhood emotional abuse, childhood physical abuse, childhood sexual abuse, duration of exploitation, post-trafficking employment status, number of unmet needs, and social support score. Variables were retained in the model if the covariate showed an association (p<0.1) with mental disorder measured at 6 months post-return. Four variables were retained and are reported above.

## Discussion

### Key findings

Over half (54.2%) of the women survivors of human trafficking in our study met DSM-IV criteria for mental disorder at an average of 6 months post-return. 35.8% of women had PTSD (alone or co-morbid), 12.5% had depression without PTSD and 5.8% had another anxiety disorder. Childhood sexual abuse, longer duration of trafficking, and post-trafficking stressors (poor social support and greater unmet needs) independently predicted mental disorder post-trafficking. These risk factors are highly compatible with the literature on PTSD and depression [17-19,32,36].

A high proportion - thirty percent - of study participants reported childhood sexual abuse; a recent systematic review reported that worldwide the prevalence of childhood sexual abuse among women ranges from 0% to 53%, with most studies reporting a prevalence of between 10% and 20% [37]. It has been proposed that pre-trauma experiences – such as childhood abuse - can act through cognitive and biological mechanisms, to increase risk of PTSD in adulthood [38]. Memories of early abuse can be reactivated by a later similar trauma and can add to the negative meaning of the new trauma [18]. Victims of childhood abuse are likely to have poor-quality memory processing when faced by a renewed trauma in adult life as they have learned to process these sorts of memories in a deficient way [18]. Disturbance of autobiographical memory is considered to be a factor underpinning the persistence of PTSD.

Characteristics of the trafficking trauma itself, such as the type and severity of violence, will be influential in delaying recovery from mental disorder. Duration of trafficking may serve as a proxy for trafficking adversity, being associated with prolonged and repeated exposure to violence, exploitation and restricted freedom [39]. Our finding that longer duration of trafficking was weakly predictive of mental disorder at an average of 6 months post-return fits with existing evidence from other PTSD populations that multiple traumas are more difficult to process than single traumas, as are traumas of longer duration, which are unpredictable, and which include sexual or other interpersonal violence [18].

It is perhaps surprising that post-trauma factors appeared so significant in predicting mental disorder, given that the nature of the trauma and psychological processes at the time of the trauma are the strongest predictors of PTSD [19]. Meta-analyses have, however, found that post-trauma social support and life stress are independent predictors of PTSD [17,19] and studies with asylum-seeking populations have shown that post-migration stressors – including discrimination, socio-economic conditions and family issues - are independently associated with mental disorder [40-42]. The cognitive model of PTSD proposes that appraisals of self and of the world following severe trauma, such as self-blame, guilt, difficulties in trusting others, and views about dangerousness of the world, are important in explaining lack of recovery from trauma [18,43,44]. Previous work has, of course, underlined the importance of life difficulties and poor social support as perpetuating factors in chronic depression [32,36]. The cognitive model of depression includes both the negative thinking styles about self and the world [45] and the recognition of predisposing, precipitating and maintaining factors in influencing the likelihood of someone having a diagnosis of depression in adulthood [46]. For the women survivors of trafficking in this study, the high rate of adverse experiences in childhood, the low level of education beyond the age of 14, the trauma as part of trafficking, and the high level of ongoing environmental stressors, would all influence onset and persistence of depression.

### Strengths and limitations

Strengths of our study included the use of methods to minimise the risk of recall bias, including through the use of standardised questionnaires, asking about behaviourally-specific examples of abuse, and using trained fieldworkers to rate unmet needs. Evidence suggests that adults are mostly accurate in recalling childhood maltreatment [47]. The risk of observer bias was minimised by diagnoses being made by a trained psychiatrist who used standardised interviews and was blind to information collected in the rest of the interview. Furthermore, the staff that provided counselling and rehabilitation were not involved in data collection.

Because the study was conducted over 18 months, it was not possible to formally test the validity of all of the study instruments. We used the official translated version of SCID used, which according to two qualified clinical academic psychiatrists, both with experience of community psychiatry and trained locally in psychiatry, had good face validity. When assessing mental disorder we did not ask about details about the exact event in relation to which women experienced PTSD, which may be a limitation of the study. However, by its nature, trafficked persons have mostly experienced severe trauma characterised by physical and sexual violence and threats while trafficked [5-8]. Criterion A1 traumatic events involving interpersonal trauma are those most likely to be associated with PTSD [30]. When assessing PTSD, the psychiatrist asked whether the participant had experienced serious harm, injury, threat of serious harm, or other very horrible experience. Subsequent questions about PTSD symptoms were asked about in relation to this event, and all of the women in the sample who met PTSD criteria answered yes to this question.

Other instruments were chosen, modified, and translated in collaboration with local informants on the basis of face validity and relevance to women survivors of human trafficking. In contrast to the CTS subscales and the CANSAS-SF, which had high Cronbach’s alpha scores, the Duke Social Support Questionnaire subscales had very low internal consistency and descriptive and regression analyses instead used the sum Duke Social Support Questionnaire score. Although the internal consistency of the sum scale was also low, its use was supported by the observed group differences. The low internal consistency may reflect the complexities of social support for these women survivors of human trafficking: participants had returned to their country of origin and received crisis support from the IOM, but may have also experienced rejection from their families and friends and may not have accessed longer-term support. Regarding affective support, for example, women tended to score the question “I get love and affection” more highly than the question “I get help when I am in trouble”, and regarding confidante support, women tended to score the question “I get chances to talk about work” more highly than “I get useful advice about important things in life.” The low internal consistency may also reflect that the high prevalence of PTSD in this sample, as PTSD frequently involves shame and avoidance of discussion [30]. It will be important to develop and validate measures of social support for future research with women survivors of human trafficking.

Our analysis of the factors that predicted post-trafficking mental disorder among women survivors of human trafficking was limited by the size of the study sample, which was dependent on the numbers of women returning to Moldova, and was restricted by the duration of study funding. Our analysis was also limited by the lack of data on women’s psychological processes during the trauma and minimal data on women’s experiences during the trafficking situation, which would have allowed us to model the role of trauma-related factors. These data were not collected for ethical reasons. Also, we did not have information on the length or type of support that women received prior to returning to Moldova or on individual mental health treatment received post-return. Emergency medical care and counselling are available in countries with IOM missions or partner organisations but it remains the case that many survivors of human trafficking have limited access to mental health services prior to repatriation. Mental health treatment in Moldova included counselling, antidepressant drugs, and, in a few cases, access to cognitive behavioral therapy (CBT).

Our sample is unlikely to be representative of all women survivors of human trafficking returning either to Moldova or elsewhere in Eastern Europe; for ethical reasons we only recruited women who had returned via the IOM assistance programme and received a crisis-intervention assessment. We do not know if the women we were unable to trace or who declined to take part had less mental disorder or were better able to cope with their social needs, or if they had worse mental health than participants. We are also unable to judge whether our findings would generalize to women who return through other means or who decline reintegration support from the IOM. Our sample is, however, representative of women in Moldova who accept post-trafficking services, in this case the standard IOM support package, which is used similarly in IOM post-trafficking services in other countries. Our findings may therefore generalize beyond Moldova to women survivors of human trafficking using IOM post-trafficking centers in other return locations (although results may differ from centers with a greater focus on follow-up mental healthcare and/or centers with a trauma focused CBT specialist). Based on our analysis, we have no reason to think that there were major differences between the 68% of those eligible who agreed to participate in research and those who could not be followed up or did not agree to participate.

### Implications

Our study comprises one of the largest surveys of women survivors of human trafficking conducted to date and is the first to diagnose psychological disorder with a validated instrument [8]. Our findings suggest the applicability of the cognitive models of PTSD and depression to this population and suggest that psychological interventions will need to take account of the chronic abuse experienced by women survivors of human trafficking both before and during their exploitation. Care plans for survivors of trafficking must be based on individual needs. Broad approaches to stabilizing physical and psychological health and attending to social needs are likely to be needed before commencing trauma-focused psychological therapy [48,49]. Psychological stabilization includes re-establishing trust in others, helping with techniques to regulate emotions and to cope with dissociation, and treating depression; moderate or severe depression may require antidepressants. Evidenced based interventions for PTSD such as trauma-focused CBT and eye movement desensitization and re-processing (EMDR) should be used for women survivors of human trafficking who are ready to talk about their trauma [45]. If women cannot engage or does not want to engage in CBT, or CBT is not available, or have ongoing severe stressors (including threats and danger), antidepressants are recommended [48]. Given the high rate of previous childhood abuse women may have personality difficulties which can mean that trauma focused CBT will take longer and require additional expertise. Longer therapy will also be required if women have troubling symptoms of PTSD in relation to several traumas, as these will need to be treated Social advocacy alongside trauma-focused CBT will be important [50].

## Conclusions

Assessment for mental disorders should be part of re-integration follow-up care for women survivors of human trafficking. Mental disorders at that time, most commonly PTSD and depression, are likely to be influenced by a range of predisposing, precipitating and maintaining factors. The combined nature of pre-trauma, peri-trauma and post-trauma factors in women survivors of human trafficking suggests that treatment of mental disorders will be challenging. Care plans for survivors of trafficking must be based on individual needs, including application of clinical guidelines for the treatment of PTSD and of depression. If person cannot engage or does not want to engage in CBT, or CBT is not available, or the woman has ongoing severe stressors, antidepressants are recommended [48]. Evidence is needed on the effectiveness of therapy for PSTD in survivors of human trafficking.

## Abbreviations

AOR: Adjusted odds ratio; APP: Assistance and protection programme; CANSAS-SF: Camberwell assessment of need short appraisal schedule; DSM-IV: Diagnostic and statistical manual of mental disorders-IV; ICD-10: International classification of disease-10; IOM: International organization for migration; PTSD: Post-traumatic stress disorder; SCID: Structured clinical interview for DSM–IV axis I disorders.

## Competing interests

The authors declare that they have no competing interests.

## Authors’ contributions

NO, MA and MP were responsible for designing the study and selecting study instruments. NO, VG, and CT collected the data. MA and MP supervised the study. MA and SO conceived and wrote the first draft of the paper. SO and MA analyzed the data. All authors read and approved the final manuscript.

## Pre-publication history

The pre-publication history for this paper can be accessed here:

http://www.biomedcentral.com/1471-244X/13/204/prepub
